# Editorial Comment from Dr Kato to A case of metastatic treatment‐emergent small cell/neuroendocrine prostate cancer with BRCA2 mutation by liver biopsy

**DOI:** 10.1002/iju5.12518

**Published:** 2022-07-28

**Authors:** Manabu Kato

**Affiliations:** ^1^ Department of Urology Aichi Cancer Center Hospital Nagoya Japan

Most urologists in clinical practice encounter suspected treatment‐emergent small cell/neuroendocrine prostate cancer (t‐SNPC) with rapid progression of disease. Post diagnosis, the treatment of these patients is difficult due to limited options; due to a poor prognosis, patients inevitably die prematurely during the course of treatment. The expression of typical markers for neuroendocrine differentiation including synaptophysin, NSE (neuron specific enolase), and chromogranin A is seen in majority of patients,^1^ while these are not expressed in other patients. However, rapid disease progression is seen in all patients. Treatment options for t‐SNPC are very limited and resistance to platinum‐based therapies develops quickly. Urologists in charge should start exploring therapies for late stage treatments for t‐SNPC; genetic diagnoses established in recent years would be a valuable resource in the discovery of new therapies. One of the genetic diagnosis methods, namely, Foundation One is being widely used in cancer hospitals across the world. The detection ratios of druggable gene mutations are low; however, there is still hope for both patients and urologists in finding available treatment options. Despite this new proposed genetic diagnostic approach, limitations existing within institutions currently prevent urologists from referring patients for genome analysis.

First, not inaccurate diagnosis of the disease state and referring patients to the genome diagnostic outpatient department and delay in consultation from the genome diagnosis department. These situations leave cancer patients at a great disadvantage when trying to develop and tailor specific treatment regimens in a timely manner. It is obvious that consultation with the genome diagnosis department is key in developing treatment regimens to improve patient care. However, there are some other limitations in Japanese hospitals. For example, a urologist cannot not directly order the exhaustive genomic testing, such as Foundation One, without an expert panel and counseling for the patients. Not all institutes have an expert panel and counseling for the patients, only few certified institutes have these systems. This difference among institutes lead a difficulty for urologists to search treatment options of aggressive prostate cancer. Currently, urologists believe that the ratio of positive detection for *BRCA* mutation is up to 10% and that there is a very low positive rate among castration‐resistant prostate cancer patients to achieve treatment goals despite the efficacy of olaparib for the inhibition of Poly (ADP ribose) polymerase for the treatment of prostate cancer cells with DNA repair defects.^2^, ^3^ Thus, prostate cancer patients in whom progression was observed even after androgen receptor axis drug treatment should be immediately examined using the above genome diagnostic procedures.

Treatment of prostate cancer cases may not be urgent compared to other clinical malignancies; however, urologists should immediately prioritize treatment for t‐SNPC patients due to its unusual rapid deviation from the “normal” prostate cancer pathology. This case presentation would be a good example for management of further cases.^4^

## Conflict of interest

The author declares no conflict of interest.

## References

[1] Akamatsu S , Inoue T , Ogawa O , Gleave ME . Clinical and molecular features of treatment‐related neuroendocrine prostate cancer. Int. J. Urol. 2018; 25: 345–51. 29396873 10.1111/iju.13526

[2] Wu J , Wei Y , Pan J *et al*. Prevalence of comprehensive DNA damage repair gene germline mutations in Chinese prostate cancer patients. Int. J. Cancer 2021; 148: 673–81. 33006389 10.1002/ijc.33324

[3] de Bono J , Mateo J , Fizazi K *et al*. Olaparib for metastatic castration‐resistant prostate cancer. N. Engl. J. Med. 2020; 382: 2091–102. 32343890 10.1056/NEJMoa1911440

[4] Naiki T , Naiki‐Ito A , Kawai T *et al*. A case of metastatic treatment‐emergent small cell/neuroendocrine prostate cancer with BRCA2 mutation diagnosed by liver biopsy. IJU Case Rep. 2022; 5: 431–5. 10.1002/iju5.12501 PMC9626340 36341200

